# Complementary Vaccination in Morocco’s Pediatric Population: A Cross-Sectional Study at Mohamed VI University Hospital, Oujda

**DOI:** 10.7759/cureus.103752

**Published:** 2026-02-16

**Authors:** Hasnae Elhaddadi, Amal Hamami, Abdeladim Babakhouya, Maria Rkain

**Affiliations:** 1 Pediatrics Department, Centre Hospitalier Universitaire Mohammed VI, Oujda, MAR; 2 Department of Pediatrics, Mohammed VI University Hospital, Oujda, MAR; 3 Faculty of Medicine and Pharmacy of Oujda, Mohammed I University, Oujda, MAR; 4 Pediatric Gastroenterology Department, Centre Hospitalier Universitaire Mohammed VI, Oujda, MAR

**Keywords:** child healthcare, complementary vaccine, national immunization program, vaccination coverage, vaccine acceptability, vaccine hesitancy, world health organization (who)

## Abstract

Introduction: Complementary vaccination (CV) in Morocco refers to vaccines administered in addition to those in the National Immunization Program (NIP). Although optional, CV plays a crucial role in preventing certain childhood diseases. This study aimed to determine the CV rate and identify factors associated with reluctance to receive CV.

Material/methods: A cross-sectional study employing descriptive and analytical methods was conducted among a sample of 450 mothers of children aged one to 16 years at the University Hospital Mohammed VI in Oujda, Morocco, over four months.

Results: All children were vaccinated according to the NIP. Most children (414; 92%) had received their obligatory vaccinations at health centers, while only 36 (8%) had received them at a private pediatrician. Only 85 children (19%) had received complementary vaccines (CVs). The hepatitis A vaccine was the most noted (89%). Mothers' knowledge of CVs is limited. Information on the names of CVs, the age of vaccine administration, the number of doses, and the benefit of the vaccine was limited among 369 mothers (82%). Mothers are often reluctant to accept CV because of a lack of knowledge about the vaccine among 351 mothers (78%), fears about vaccine safety among 279 mothers (62%), the unimportance of the vaccine among 256 mothers (57%), the non-obligatory nature of the vaccine among 234 mothers (52%), the sufficiency of compulsory NIP vaccines among 225 mothers (50%), the non-availability of the vaccine at the health center among 220 mothers (49%) and the high cost of the vaccine among 202 mothers (45%). Statistical analysis showed that multiparity (*OR* = 1.02; *95% CI*: 1.21-2.26) and university-level education (*OR *= 1.52; *95% CI*: 1.23-3.23) were significantly associated with CV acceptance. Having an only child (*OR *= 1.56; *95% CI*: 1.11-2.07), low socioeconomic status (*OR* = 1.54; *95% CI*: 1.12-2.17), and lack of medical coverage (*OR* = 1.20; *95% CI*: 0.99-2.01) were significantly associated with reluctance to undergo CV. The results of the chi-square test revealed that the proportion of acceptability of supplementary vaccination increased with younger maternal age (χ2 = 19.74, p < 0.001*), higher maternal education (χ2 = 24.05, p < 0.001*), availability of health coverage (χ2 = 16.66, p < 0.001*) and higher family socioeconomic level (χ2 = 19.75, p < 0.001*). In the case of mothers whose children had received CV, all mothers rated their experience of vaccination with CVs as good.

Conclusion: The results of our study highlighted a low rate of CV, as well as the various factors influencing this type of vaccination. In Morocco, CVs are available in the private sector, but the demand for and acceptability of these vaccines seem to depend on informing parents and raising the awareness of healthcare personnel. Government efforts are needed to tackle the challenges identified in our study.

## Introduction

Vaccination is one of the most effective interventions for the prevention of infectious diseases [1]. The use of vaccines has contributed to public health advances in the 20th century, including the prevention of millions of deaths and significant reductions in the incidence and mortality of vaccine-preventable diseases (VPDs), such as measles, polio, and diphtheria [2]. Many countries have implemented National Immunization Programs (NIPs) to demonstrate leadership in public health, reduce epidemics, ensure the protection of individuals, communities, and vulnerable populations, and reduce hospital admissions and associated costs [3].

In Morocco, the first vaccination campaigns date back to the early 1960s, and the Expanded Program on Immunization (EPI) was launched in 1981. The EPI was restructured into the NIP six years later. Today, the Moroccan NIP includes vaccination against 12 VPDs. It is free of charge and available in dispensaries and public health centers [4]. The NIP refers to the planned vaccination of the population with different vaccines and vaccination procedures determined by the government. However, NIP progress differs from country to country [5]. On a global scale, Japan established the National Vaccination Law in 1948 to recommend vaccination and prevent 12 VPDs. Currently, Japan's NIP contains 12 vaccines to prevent 16 VPDs [6]. In 1961, the UK published its first national routine immunization schedule. Currently, this program contains 17 vaccines, aimed at vaccinating a targeted population: children, pregnant women, the elderly, and adults [7]. In 1978, China launched its National Vaccination Program to control the spread of infectious diseases, aiming to control six diseases with four vaccines. After several modifications, 16 vaccines were included in China's NIP [8].

In addition to NIP vaccines, other vaccines not included in the NIP are recommended by the World Health Organization (WHO), administered voluntarily and self-financed by citizens [9]. As an effective complement to NIP vaccines, complementary vaccines (CVs) are playing an increasing role in the prevention of specific or emerging diseases. Unlike NIP vaccines, which are largely financed and accessible thanks to public policies, CVs are often aimed at targeted pediatric populations or those with specific needs, at-risk groups, or individuals exposed to particular pathologies [10]. In Morocco, the main vaccines considered complementary are varicella, viral hepatitis A, influenza, and meningococcal.

Hepatitis A virus (HAV) infections continue to represent a significant burden of disease worldwide, causing an estimated 200 million infections, 30 million symptomatic illnesses, and 30,000 deaths per year [11]. Safe, effective hepatitis A vaccines have been available since the early 1990s. Developed for individual prophylaxis, HAV vaccines are now increasingly used to control hepatitis A in endemic areas [12]. The HAV vaccine is recommended by the WHO and is included in several NIPs such as China, the USA, and Russia [13]. In Morocco, the hepatitis A vaccine is not included in the NIP, although it is recommended from the age of 1 and is administered in two doses, usually six to 12 months apart. Although chickenpox is generally a benign, self-limiting disease, complications can occur. In 1998, the WHO recommended varicella vaccination in countries where the disease represented a significant public health burden [14]. Despite consistent data on vaccine immunogenicity and efficacy (EV), questions remain about the introduction of a universal vaccine. Currently, the number of countries with a universal vaccine is increasing [15]. Varicella vaccine is not included in Morocco's NIP, but it is available in the private sector and recommended as a supplementary vaccination from the age of one. It is administered in two doses, usually six to 12 months apart.

The WHO considers influenza to be a major health threat in today's world. For example, seasonal influenza alone is responsible for three to five million cases of severe illness and 250,000 to 500,000 deaths worldwide annually [16]. Most influenza-related deaths occur in the most vulnerable populations, namely, very young children, the elderly, and patients with chronic diseases. Despite the severity of influenza and the availability of safe vaccines, low influenza vaccination rates remain a challenge worldwide and contribute to the burden of disease [17]. In our context, Morocco is actively working to expand its influenza vaccination policy to cover high-risk groups, as recommended by the WHO [18].

Invasive meningococcal disease (IMD) is a rare but serious infection caused by *Neisseria meningitidis* and associated with high morbidity and mortality [19]. Although the incidence of IMD is highest in infants, a second peak occurs in adolescents and young adults. The incidence of IMD and the main meningococcal serogroups responsible for the disease vary worldwide, with groups A, B, C, W135, Y, and X responsible for the majority of the disease. Epidemiological data have guided the development of meningococcal vaccines to reduce the disease burden of IMD [20]. In Morocco, where the epidemiological profile of meningococcal meningitis is dominated by serogroup B, which accounts for up to 70% of confirmed cases of *Neisseria meningitidis*, depending on the year, bivalent AC or tetravalent ACYW135 polysaccharide vaccines have been used for several years as part of the national meningitis control program.

Existing studies on CV are rare, reflecting a lack of in-depth exploration in this subject. To our knowledge, our study constitutes the first analysis dedicated to CV in Morocco, thus contributing to filling a gap in the scientific literature and shedding light on a public health issue still little explored in this context. Our study aims to carry out a global survey on CV in Morocco, and more specifically in the Oriental region, to determine the rate of CV, assess mothers' knowledge on the subject, and identify the socio-demographic factors associated with reluctance towards these CVs, to provide strategic suggestions for extending and improving the Moroccan government's policy of vaccines outside the NIPs.

## Materials and methods

Study design

This was a cross-sectional study, adopting both a descriptive and analytical approach. It was conducted over 4 months, from August to November 2024. The study took place at the Mohammed VI University Hospital of Oujda, Morocco, and combined an observational and descriptive approach to the results obtained from completed questionnaires with an analytical approach using a statistical program.

Study sample

The Oriental region, whose capital is the town of Oujda-Angad Prefecture, is located in eastern Morocco, and its population is estimated at around 2,600,440 in 2024, according to projections by the High Commission for Planning. This population is divided between rural and urban areas, with significant differences in lifestyle, cultural level, and socioeconomic conditions. Mothers of children hospitalized in our department or consulting pediatric emergency departments were invited to participate in our study. The study population consisted of 450 mothers. The inclusion criteria were mothers of children aged one to 16 who agreed to take part in our study. We specifically chose to include mothers rather than fathers or other accompanying caregivers because, in the Moroccan context, mothers are generally the primary caregivers who accompany their children to vaccination appointments and medical consultations. They are therefore the most knowledgeable about their child’s vaccination history, including both mandatory and CVs, which helps ensure the accuracy and completeness of the collected information.

Data collection methods

Data collection was structured using a questionnaire (see Appendix). The first part of the data collected the sociodemographic characteristics of the mothers (age, level of education, socioeconomic level, family situation, living environment, number of children, type of health coverage, etc.) and their children (age, sex, pathological antecedents, vaccination according to the NIP, regular follow-up with a pediatrician or family doctor, supplementary vaccination). Vaccination status was determined from the children's health records. In the second part of the data, we sought to assess mothers' knowledge of CV (names and types of vaccines, costs, availability, benefits, sources of information, etc.). The third part of the data focused on factors related to acceptability, reluctance, or hesitancy to have their children vaccinated with CVs, which are not included in the NIP. The study was conducted in compliance with ethical principles: informed consent was obtained from participants, and anonymity and confidentiality of data were strictly guaranteed.

Interpretation of results and statistical analysis

Data were encoded using IBM SPSS Statistics for Windows, Version 24.0 (released 2016, IBM Corp., Armonk, NY). After checking responses, questionnaires with missing or contradictory data were excluded (number = 30 questionnaires). Descriptive statistics were generated for all variables. Qualitative variables (gender, level of education, etc.) were calculated and presented in absolute and relative frequencies (in percentages) in the form of tables or diagrams (bars, sectors). Quantitative variables (age, etc.) were presented as mean, median, standard deviation, minimum, and maximum. The existence of associations and comparisons between categorical variables was demonstrated using the Chi2. Logistic regressions were carried out to identify the factors involved in the acceptability, reluctance, or hesitancy of children's supplementary vaccination. Univariate logistic regression models were applied to obtain the odds ratio (OR) with a 95% confidence interval (CI). For multiple logistic regression, variables with a p-value > 0.2 were excluded. The corresponding p-value for the various statistical tests was considered significant for p < 0.05.

## Results

After excluding questionnaires with missing or contradictory data, 450 questionnaires were analyzed.

Mothers' sociodemographic characteristics

The average age of the mothers was 45 years (min: 22; max: 51). Low socioeconomic status was noted in 279 families (62%). A total of 323 mothers (72%) had a schooling level no higher than secondary school, while 103 mothers (13%) were analphabetic. Meanwhile, 374 mothers (83%) were housewives, while only 45 mothers (10%) were civil servants. One-hundred twenty six mothers (28%) lived in the context of family conflict (divorce - marital problems). "Only child” status was noted in 158 families (35%), while the rest of the mothers had two children, 189 (42%), or more than two children, 103 (23%). The living environment was divided between urban 324 (72%) and rural 126 (28%) areas. A total of 382 mothers (85%) had compulsory health insurance coverage (Table 1).

**Table 1:** Sociodemographic characteristics of mothers.

Variables		Number (percentage)
Age of mothers	<20 years	03 (01%)
	20-40 years	327 (73%)
	>40 years	120 (26%)
Socio-economic level	Favorable	171 (38%)
	Unfavorable	279 (62%)
Educational level	Illiterate	103 (23%)
	Primary	90 (20%)
	Secondary	130 (29%)
	University	127 (28%)
Health coverage	Compulsory health insurance	382 (85%)
	No health coverage	68 (15%)
Mothers' professions	Housewife	374 (83%)
	State employee	45 (10%)
	Free civil servant	31 (7%)
Living environment	Rural	126 (28%)
	Urban	324 (72%)
Number of children	One child	158 (35%)
	Two children or more	292 (65%)

Children's sociodemographic characteristics

The average age of the children was 6.3 years (min: one year; max: 15 years seven months). The most common age range was eight to 12 years (54%). Females were the most represented, 310 (69%). All children attended school (100%). About pathological antecedents, 12 children (2.9%) had medical-surgical antecedents: asthma (six children), appendectomy (three children), celiac disease (one child), hypothyroidism (one child), and death in the fraternity in the context of hepatic encephalopathy secondary to hepatitis A. Only 99 children (22%) had regular follow-up with a private pediatrician (Table 2). 

**Table 2:** Children's sociodemographic characteristics.

Variables		Number (percentage)
Age of the child	1 year–5 years	135 (30%)
	8–12 years	243 (54%)
	>12 years	72 (16%)
Gender	Female	310 (69%)
	Male	140 (31%)
Schooling	Yes	450 (100%)
	No	00 (00%)
Regular follow-up with a pediatrician or family doctor	Yes	99 (22%)
	No	351 (78%)
Vaccination	Vaccinated according to PNI	450 (100%)
	Imcompleted-vaccination	00 (00%)
	Not vaccinated	00 (00%)
Complementary vaccination	Yes	85 (19%)
	No	365 (81%)
Medical history	No antecedents	438 (97%)
	Asthma	06 (1.3%)
	Celiac disease	01 (0.2%)
	Hypothyroidism	01 (0.2%)
	Appendectomy	03 (0.7%)
	Death in the family due to post-HVA hepatic encephalopathy	01 (0.2%)

Vaccination data

All children were vaccinated according to the NIP. A total of 414 children (92%) had received their obligatory vaccinations at health centers, while only 36 children (08%) had received them at a private pediatrician. Only 85 children (19%) had received CVs, including 75 children (89%) with the hepatitis A vaccine and 10 children (11%) with the varicella vaccine. No patients received influenza or meningococcal vaccines. No adverse events were reported by the mothers, apart from post-vaccination fever. In all cases, CV was recommended by the pediatrician. Vaccines were purchased by the family in all cases and administered in private pediatric practices. No unusual adverse events were reported by the mothers (Table 3).

**Table 3:** Vaccination data of children.

Vaccination data of children	Number (percentage)
Vaccination according to the National Immunization Program (NIP)	450 (100%)
Vaccination received at the health center	414 (92%)
Vaccination received from a private pediatrician	36 (08%)
Complementary vaccination received	85 (19%)

Mothers' knowledge of CV

Of the 450 participants, 310 mothers (69%) did not know that vaccines are divided into vaccines included in the NIP and vaccines not included in the NIP, 120 mothers (26%) knew that vaccines not included in the NIP were voluntary vaccines, which were administered at their own expense, and 89 mothers (21%) thought that vaccines not included in the NIP were unnecessary as they were not compulsory (Table 4).

**Table 4:** Mothers' knowledge of complementary vaccination.

Mothers' knowledge of complementary vaccination	Yes (Number / Percentage)	No (Number / Percentage)
Vaccines are divided into vaccines included in the NIP and vaccines not included in the NIP.	310 (69%)	140 (31%)
Vaccine not included in the NIP were a voluntary vaccine.	120 (26%)	330 (74%)
Vaccine not included in the NIP were administered at their own expense.	120 (26%)	330 (74%)
Vaccines not included in the NIP were unnecessary as they were not compulsory.	89 (21%)	361 (79%)

Our study showed that mothers' knowledge of CVs is limited. Information on the names of CVs, the age of vaccine administration, the number of doses, and the benefit of the vaccine was limited among 369 mothers (82%). In addition, 10 mothers (02%) said they had received information about vaccines from a pediatrician, and via the Internet. 

Factors influencing CV

Our study showed that mothers are often reluctant to accept CV because of a lack of knowledge about the vaccine among 351 mothers (78%), fears about vaccine safety among 279 mothers (62%), the unimportance of the vaccine among 256 mothers (57%), the non-obligatory nature of the vaccine among 234 mothers (52%), the sufficiency of compulsory NIP vaccines among 225 mothers (50%), the non-availability of the vaccine at the health center among 220 mothers (49%) and the high cost of the vaccine among 202 mothers (45%) (Table 5).

**Table 5:** Factors influencing complementary vaccination among the participants. NIP: National Immunization Program

Factors	Number (Percentage)
The lack of knowledge about the vaccine	351 (78%)
The fears about vaccine safety	279 (62%)
The unimportance of the vaccine	256 (57%)
The non-obligatory nature of the vaccine	234 (52%)
The sufficiency of compulsory NIP vaccines	225 (50%)
The non-availability of the vaccine at the health center	220 (49%)
The high cost of the vaccine	202 (45%)

Statistical analysis of the data showed that multiparity (OR = 1.02; 95% CI: 1.21 to 2.26), university level of education (OR = 1.52; 95% CI: 1.23-3.23), regular follow-up with a private practice pediatrician (OR = 1.19; 95% CI: 1.11-2.07), having a family member who is a healthcare professional (OR = 1.99; 95% CI: 1.51-2.87), and using social media (OR = 1.49; 95% CI: 1.01-2.07) were significantly associated with CV acceptance. On the other hand, having an only child (OR = 1.56; 95% CI: 1.11-2.07), being a housewife (OR = 1.12; 95% CI: 1.09-1.37), low socioeconomic level (OR = 1.54; 95% CI: 1.12-2.17), low level of education (OR = 1.17; 95% CI: 1.01-1.34), and unavailability of medical coverage (OR = 1.20; 95% CI: 0.99-2.01) were significantly associated with reluctance and hesitancy towards CV (Table 6).

**Table 6:** Factors significantly associated with acceptance and hesitancy toward CV: ORs and 95% CI. CV: complementary vaccination, OR: odds ratio, CI: confidence interval

Factor	Odds Ration (OR)	95% CI	Association
Multiparity	1.02	1.21 – 2.26	Acceptance
University level of education	1.52	1.23 – 3.23	Acceptance
Regular follow-up with private pediatrician	1.19	1.11 – 2.07	Acceptance
Having a family member who is a healthcare professional	1.99	1.51 – 2.87	Acceptance
Use of social media	1.49	1.01 – 2.07	Acceptance
Having an only child	1.56	1.11 – 2.07	Hesitancy
Being a housewife	1.12	1.09 – 1.37	Hesitancy
Low socio-economic level	1.54	1.12 – 2.17	Hesitancy
Low level of education	1.17	1.01 – 1.34	Hesitancy
Lack of medical coverage	1.20	0.99 – 2.01	Hesitancy

Acceptability of vaccines not included in the NIP

The results revealed that 313 participants (69.5%) had a positive intention to vaccinate their child with vaccines not included in the NIP if they were made aware and informed about the importance of CVs. Acceptability would increase to (86.3%) if these vaccines were covered or reimbursable by health insurance at 100% or offered at the health center free of charge. The results of the chi-square test revealed that the proportion of acceptability of supplementary vaccination increased with younger maternal age (χ2 = 19.74, p < 0.001*), higher maternal education (χ2 = 24.05, p < 0.001*), availability of health coverage (χ2 = 16.66, p < 0.001*) and higher family socio-economic level (χ2 = 19.75, p < 0.001*). In the case of mothers whose children had received CV, all mothers rated their experience of vaccination with CVs as good. Perceived convenience (χ2 = 8.07, p < 0.001*) and vaccination satisfaction (χ2 = 5.95, p < 0.001*) showed a significant relationship with the acceptability of vaccines not included in the NIP for their children (Table 7).

**Table 7:** Chi-square analysis of sociodemographic determinants influencing supplementary vaccination acceptability.

Factor	Trend observed	Chi-square (χ²)	p-value	Significance
Maternal age	Acceptability increased with younger age	19.74	<0.001*	Significant
Maternal education level	Acceptability increased with a higher level	24.05	<0.001*	Significant
Availability of health coverage	Acceptability higher with coverage	16.66	<0.001*	Significant
Family socio-economic level	Acceptability increased with a higher level	19.75	<0.001*	Significant

## Discussion

Our study used a regional sample to estimate the rate of CV and to examine acceptability and hesitancy toward vaccines not included in the NIP among mothers of children aged one to 16 years, as well as factors associated with mothers' intention to vaccinate their children with CVs. Understanding these factors is important to provide a scientific and theoretical basis for promoting the use of vaccines and strengthening the prevention and control of infectious diseases. 

Rate of CV 

As in our study, research in the literature has highlighted low vaccination coverage rates for vaccines not included in NIPs. The study by Ji M et al. showed that infants in Shanghai had significantly lower vaccination coverage for these vaccines than those included in the NIP. At 15 months, only 68% of children had received a dose of *Haemophilus influenzae* b (Hib) vaccine, 13% a dose of pneumococcal conjugate vaccine (PCV), and 52% a dose of rotavirus vaccine (RVV). However, it is recommended to administer three doses of Hib before the age of six months and four doses of PCV and RVV before the age of five months [21]. The study by Choe YJ et al. found that all NIP vaccines achieved higher coverage than vaccines not included in the NIP and that booster dose coverage rates for vaccines not included in the NIP were significantly lower than those for the NIP vaccine series [3]. An investigative study by Jiang M et al. investigated the vaccination rates of four vaccines not included in the NIP (*Haemophilus influenzae *type b vaccine, human papillomavirus vaccine, pneumococcal conjugate vaccine, and rotavirus vaccine) and the main obstacles faced by health systems, health care providers and caregivers in increasing this coverage, this study found a low rate of these four vaccines compared with the NIP vaccines [22].

Between hesitation and acceptance of CV

In our study, lack of knowledge about the vaccine, fears about vaccine safety, the non-obligatory nature of the vaccine, the sufficiency of the compulsory vaccines in the NIP, the unavailability of the vaccine at the health center, and the high cost of the vaccine are all reasons reported by mothers for not having their children vaccinated with vaccines not included in the NIP. In the literature, parents who are reluctant to be vaccinated are much more numerous than those who refuse to be vaccinated. The reasons for vaccine hesitancy are complex and encompass much more than a simple lack of knowledge [23]. The study by Ji M et al [21], which examined the relationship between vaccine hesitancy and uptake, has helped us to understand how government regulations can have an impact on vaccine coverage, even in the presence of widespread hesitancy. Comparing the impact of vaccine hesitancy in NIP and non-NIP vaccines, adding vaccines to the NIP list attenuates the impact of parental hesitancy. This may require expanding the NIP list to include high-burden but low-adoption vaccine-preventable diseases in the future. Worldwide, there is widespread and consistent support for obligatory vaccines [24]. The meta-analysis by Jiang M et al. showed that financial barriers were mainly related to the high price of vaccines, given the low-income level of citizens. Fourteen of the 19 articles included in the meta-analysis indicated that the current price of vaccines not included in the NIP in China was generally too expensive for the average family. Moreover, there is a difference in price between locally produced and imported vaccines [22].

Role of healthcare professionals

Social inequalities in awareness contribute to mothers' lack of knowledge about CV and its benefits. Previous studies have shown a strong positive correlation between the recommendation behavior of public health workers and children's vaccination status. Physicians providing information on CVs increase families' knowledge on the subject [25]. The Canadian study by Donna M MacDougall et al. focused on estimating the vaccination rate of the Canadian pediatric population against rotavirus, a vaccine that is optional and not included in the Canadian NIP, and found that families informed by healthcare professionals have more positive attitudes toward vaccines, their safety, efficacy, and risks. They assess the seriousness and risk of potential illness for the child [26]. In Turkey, the number of studies conducted on this subject reflects doctors' limited knowledge of CVs [27]. In a study by Elitok et al., the rates of pediatricians' and family doctors' adequate knowledge of non-NIP vaccines in Turkey among adolescents were reported at 10% and 5.4%, respectively, with statistically significant differences [28]. Furthermore, our study revealed that recognition of the safety, efficacy, and necessity of CVs was associated with acceptance by mothers. Mothers were often asked to decide whether or not their children should receive these vaccines. Consequently, their understanding and knowledge of vaccines not included in the NIP were significantly related to their acceptability. 

Factors influencing CV

Statistical analysis of the data from our study showed that having an only child, being a housewife, low socioeconomic status, low level of education, and unavailability of medical coverage were significantly associated with reluctance and hesitancy towards CV. In the literature, the study by Ji M. et al. found lower coverage among rural versus urban residents. In addition to residence status, people with a higher level of education were more likely to have their children vaccinated with vaccines not included in the NIP [21]. In the same vein, the study by Wang X et al. was based on an interview with 30 rural parents in a village in central China to investigate their knowledge of vaccines not included in the NIP and their intention and behavior regarding CV. It showed that a low educational level has a strong influence on the decision to vaccinate [9].

Multivariate analysis of the study by Wu l et al. showed that income level was significantly correlated with willingness to obtain CVs for children and that parents with higher family income had greater acceptance of vaccines not included in NIPs for their children [10]. In the same study, parents with higher levels of education were associated with greater acceptance of CVs. Several other studies have also shown that there is a significantly positive correlation between parents' level of education and acceptance of non-NIP vaccines [29].

Limitations and future research

This study is of great importance for building a structural model of the decision-making process regarding vaccines not included in the NIP, and for providing practical implications to guide CV policy in Morocco. Although the sample selected for the survey appears limited and cannot comprehensively represent the Moroccan population, it remains sufficiently reliable to assess the various factors influencing parents' intention to use CVs and to reflect regional differences in Morocco in terms of economic development, public policy, and health literacy. This study used purposive sampling and interviewed only mothers of children aged 0-16 years, which may lead to errors and biases in the results. As the mothers interviewed in this study were, in most cases, housewives and illiterate, and most had limited knowledge of CVs, relevant explanations and interpretations were often required when researchers contacted them, which may lead to possible response bias on the part of the researchers. Further studies in the future could expand the research to include fathers in the survey and use a higher number of participants with more random sampling to increase the generalizability of the results. 

## Conclusions

In our study, we identified a low rate of CV, as well as the various factors influencing this type of vaccination. In our country, CVs are available in the private sector, but the demand for and acceptability of these vaccines seem to depend on informing parents and raising the awareness of healthcare personnel. We, therefore, suggest using various communication media to reach as many professionals and parents as possible, organizing national awareness campaigns in schools and health centers, and supporting all programs and actions already in place or being launched by the Ministry of Health and Social Protection to introduce these vaccines into the Moroccan NIP. 

## Appendices

**Table 8:** Questionnaire used in our study.

Mothers' socio-demographic characteristics Please find below the questions you are invited to answer. Select the box(es) corresponding to your choice.		
Age of mothers	……………………. Years	
Socio-economic level	Favourable	
	Unfavorable	
Educational level	Illiterate	
	Primary	
	Secondary	
	University	
Health coverage	Compulsory health insurance	
	No health coverage	
Mothers' professions	Housewife	
	State employee	
	Free civil servant	
	Other answer ………………	
Living environment	Rural	
	Urban	
Number of children	1 child	
	2 children	
	More than 2 children	
Children's socio-demographic characteristics Please find below the questions you are invited to answer. Select the box(es) corresponding to your choice.		
Age of the child	1 year – 5 years	
	8– 12 years	
	>12 years	
Gender	Female	
	Male	
Schooling	Yes	
	No	
Regular follow-up with a pediatrician or family doctor	Yes	
	No	
Medical history	…………………………………………	
Vaccination data Please find below the questions you are invited to answer. Select the box(es) corresponding to your choice.		
Has your child received his mandatory vaccination?	Vaccinated according to PNI	
	Imcompleted-vaccination	
	Not vaccinated	
where you received the compulsory vaccination?	Public health center	
	Private pediatric practice	
Has your child received any complementary vaccinations? if so, what type of complementary vaccine?	Yes ………………………….	
	No	
Where you received the complementary vaccination?	Public health center	
	Private pediatric practice	
Who paid for the complementary vaccination?	The family	
	Health coverage	
	Other response ………………………………………………………………………………………………	
Have you noticed any unusual side effects from the complementary vaccines?	Yes	
	No	
Mothers' knowledge of complementary vaccination Please find below the questions you are invited to answer. Select the box(es) corresponding to your choice.		
Did you know that there are compulsory vaccines included in Morocco's National Immunization Program (NIP) and complementary vaccines not included in the NIP?	Yes	No
Did you know that complementary vaccination is voluntary?	Yes	No
Did you know that complementary vaccines are administered at your own expense?	Yes	No
What complementary vaccines are available in Morocco? For each vaccine, indicate age of administration, number of doses and benefits.	Yes	No
Have you received any information about complementary vaccination? If so, what are your sources?	Yes	No
Factors influencing complementary vaccination Please find below the questions you are invited to answer. Select the box(es) corresponding to your choice.		
What are your reasons for being reluctant about complementary vaccination?	Lack of knowledge about the vaccine	
	Fears about vaccine safety	
	The unimportance of the vaccine	
	The non-obligatory nature of the vaccine	
	The sufficiency of compulsory NIP vaccines	
	The non-availability of the vaccine at the health center	
	The high cost of the vaccine	
	Other response ……………………………………………………………………………………………………………………………………………………………………………………….	
Acceptability of vaccines not included in the national immunization program Please find below the questions you are invited to answer. Select the box(es) corresponding to your choice.		
Would you have a positive intention to vaccinate your child with vaccines not included in the NIP if you are aware and informed of the importance of this type of vaccination?	Yes	
	No	
Would you have a positive intention of vaccinating your child with vaccines not included in the NIP if they were covered or reimbursed by health insurance at 100% or offered free of charge at the health center?	Yes	
	No	
How do you find your experience with complementary vaccination?	Good	
	Bad	
